# Predicting Protein–Protein Interactions Between Rice and Blast Fungus Using Structure-Based Approaches

**DOI:** 10.3389/fpls.2021.690124

**Published:** 2021-07-23

**Authors:** Cunjian Zheng, Yuan Liu, Fangnan Sun, Lingxia Zhao, Lida Zhang

**Affiliations:** Department of Plant Science, School of Agriculture and Biology, Shanghai Jiao Tong University, Shanghai, China

**Keywords:** protein-protein interactions, rice, blast fungus (*Magnaporthe oryzae*), protein structure, machine learning

## Abstract

Rice blast, caused by the fungus *Magnaporthe oryzae*, is the most devastating disease affecting rice production. Identification of protein–protein interactions (PPIs) is a critical step toward understanding the molecular mechanisms underlying resistance to blast fungus in rice. In this study, we presented a computational framework for predicting plant–pathogen PPIs based on structural information. Compared with the sequence-based methods, the structure-based approach showed to be more powerful in discovering new PPIs between plants and pathogens. Using the structure-based method, we generated a global PPI network consisted of 2,018 interacting protein pairs involving 1,344 rice proteins and 418 blast fungus proteins. The network analysis showed that blast resistance genes were enriched in the PPI network. The network-based prediction enabled systematic discovery of new blast resistance genes in rice. The network provided a global map to help accelerate the identification of blast resistance genes and advance our understanding of plant–pathogen interactions.

## Introduction

Rice blast, caused by the fungus *Magnaporthe oryzae*, is the most devastating disease affecting rice production. Due to the availability of both genome sequences and the accessibility of molecular genetic tools, the pathosystem between rice and blast fungus has become a model system for studying plant–pathogen interactions (Dean et al., 2005; International Rice Genome Sequencing Project., 2005). Although the molecular mechanisms of the plant immune system have been extensively investigated over the past decade, many aspects of the overall resistance picture remain poorly understood (Meng et al., 2019).

Protein–protein interactions (PPIs) play a critical role in molecular recognition between plants and pathogens. Identification of these PPIs is important for understanding the underlying molecular mechanisms against pathogen infection in plants. Experimental methods have been used to identify plant–pathogen PPIs (Mukhtar et al., 2011; Weßling et al., 2014; Cao et al., 2019), but the available interaction data are still far from depicting global maps of plant–pathogen interactions (Ammari et al., 2016). Only a few experimentally verified PPIs between rice and blast fungus have been reported in the individual studies, which is insufficient to elucidate the molecular mechanisms leading to disease resistance in rice (Kanzaki et al., 2012; Cesari et al., 2013).

To complement experimental methods for identifying PPIs, many computational methods have been developed to accelerate the discovery of PPIs (Tanwar and George Priya Doss, 2018). Most available computational methods such as interolog mapping (Matthews et al., 2001), domain-based inference (Deng et al., 2002), gene fusion (Marcotte et al., 1999), phylogenetic similarity (Pellegrini et al., 1999), and gene co-expression [28] are primarily focused on determining PPIs within a single organism. Some of these methods, such as interolog and domain-based inference, have also been applied to the interspecies PPI field and lead to the discovery of important biological insights in plant–pathogen interactions (Li et al., 2012; Sahu et al., 2014; Yang et al., 2019).

Recently, computational methods using structural information to predict PPIs have gained much attention due to the rapid growth of protein three-dimensional (3D) structures (Zhang et al., 2012; Burley et al., 2017). Rather than sequence-based methods, structure-based approaches could reveal the structural details of protein interactions (Mariano and Wuchty, 2017). However, the structure-based methods require the 3D characterization of each protein and therefore suffer from low coverage of the proteome. Predicting protein interactions based on homology-modeled structures might be a solution to this problem, which enabled the use of protein structural information on a genome-wide scale (Zhang et al., 2016; Liu et al., 2017; Zhao et al., 2019).

In this study, we presented a computational framework for predicting plant–pathogen PPIs based on structural information. Performance assessment showed that the structure-based method was powerful in discovering PPIs between plants and pathogens. Furthermore, we used the structure-based method to generate a global PPI network between rice and blast fungus proteins, which provided a valuable reference for systems understanding of plant and pathogen interactions.

## Materials and Methods

### Data Sources

All experimentally verified host–pathogen interactions were collected from the Host–Pathogen Interaction Database (https://hpidb.igbb.msstate.edu/index.html) (Ammari et al., 2016). A total of 10,148 host–pathogen PPIs with homology models and experimental structures were used as the positive reference dataset, while the interacting protein pairs between hosts and pathogens were randomly shuffled to form the negative reference dataset.

The genome of *M. oryzae* strain 70–15 was downloaded from Ensembl Genomes (http://fungi.ensembl.org/Magnaporthe_oryzae/Info/Index), and the genome of *Oryza sativa* ssp. *japonica* cv. Nipponbare was downloaded from the MSU Rice Genome Annotation Project Database (http://rice.plantbiology.msu.edu/). A total of 38,864 non-transposable element protein sequences were identified in the rice genome.

### Identification of Membrane and Secreted Proteins in Blast Fungus

The membrane proteins containing one or more transmembrane helices were predicted by using TMHMM (Möller et al., 2001). The putatively secreted proteins were identified when the protein containing a signal peptide was predicted by using SignalP-5.0 (Almagro Armenteros et al., 2019) and the extracellular localization of the protein was predicted by using WoLF PSORT (Horton et al., 2007).

### Homology Modeling of Protein Structures

Homology models of proteins were built by using ModPipe (Pieper et al., 2014). The homology structure with the highest ModPipe quality score was selected for each protein according to the previously described criteria (Zhang et al., 2016; Liu et al., 2017).

### Structural Template for the Interaction Model

A total of 157,771 protein complexes involving 328,671 chains were collected from the Protein Data Bank (PDB) (Burley et al., 2017). The chain–chain binary interfaces of protein complexes were generated by PIBASE with an interatomic distance cutoff of 6.05 Å (Davis and Sali, 2005).

### Structure-Based Features

Structural alignment was used to find the closest PDB chains of homology models using TM-align with the cutoff score of 0.4 (Zhang and Skolnick, 2005). The interaction model of protein pair was created by superimposing the homology structures on their corresponding chains in the closest PDB template complex. Four structural features, including structural similarity (i.e., TM-score) and structural distance (i.e., root mean square deviation, RMSD) between protein homology models and their corresponding chains in the template, as well as the number and fraction of interacting residue pairs in the template that were preserved in the interaction model, were calculated for the prediction of PPIs. The detailed method for structural features refers to the previous study (Zhang et al., 2016).

### Prediction of Interolog and Domain-Based PPIs

The potential PPIs were predicted using interolog mapping. Each protein was blasted against the experimentally determined PPI datasets to identify homologs with an *E*-value of <10^−5^, a sequence identity of >45%, and an aligned sequence coverage of >50%. The experimentally determined PPIs were derived from the BioGRID (Oughtred et al., 2021), IntAct (Orchard et al., 2014), MINT (Calderone et al., 2020), DIP (Salwinski et al., 2004), and BIND (Alfarano et al., 2005) databases (Supplementary Table 1).

The domains of each protein were identified by PfamScan against the Pfam database (Mistry et al., 2021). The interacting domains were identified based on the host–pathogen PPIs and collected from the 3 did (three-dimensional interacting domains) database (Mosca et al., 2014). When an interacting domain pair were present in two proteins, the two proteins were expected to interact with each other.

### Performance Evaluation of Predictive Models

To evaluate model performance across different host–pathogen systems, we trained on the dataset in the training host–pathogen systems and evaluated the performance on the test host–pathogen system. In other words, the entire training dataset was partitioned into two parts, i.e., test set and training set. Test set contained dataset from one host and all its pathogens, and training set included all datasets related to the other remaining hosts and pathogens. We used the test set from seven hosts (i.e., *Mus musculus, Arabidopsis thaliana, Rattus norvegicus, Aedes aegypti, Bos taurus, Sus scrofa*, and *Gallus gallus*) and their pathogens to evaluate the model performance across host–pathogen systems, respectively. For each test host–pathogen system, the process was performed 10 times with the different negative training sets.

The 10-fold cross-validation method was used to evaluate the performance of different models. The training dataset was randomly divided into 10 subsets. Nine of them were combined to train the model, and the remaining one was used to test the model. The progress was repeated 10 times with the different negative training sets, and the final result was the average performance of the 10 replicates. True positive rate (TPR) = TP/(TP + FN), false positive rate (FPR) = FP/(FP + TN), precision = TP/(TP + FP), and F1 score = 2 × (precision × recall)/(precision + recall) were used to evaluate the prediction performance.

### Prediction of PPIs Between Rice and Blast Fungus

We built the rice–blast fungus PPI classifier using the random forest from the scikit-learn library in Python. The interaction probability of each protein pair was computed using the optimized model. The protein pair with a probability greater than the threshold of 0.5 was considered to interact with each other.

### Identification of Avirulence Genes in Blast Fungal Genome

Gene sequences of avirulence effectors were extracted from the GenBank database (Meng et al., 2019). All predicted coding sequences of the blast fungal genome were then searched against the local avirulence sequence database to identify matches to the cloned genes. The parameters used for the sequence similarity search were ≥95% identity and 80% coverage of the avirulence effector genes.

### Analysis of Functional and Pathway Enrichment

Functional enrichment of rice genes was analyzed by using agriGO (Tian et al., 2017) by comparing the reference gene dataset of the rice genome with their False Discovery Rate (FDR) values. The pathway enrichment analysis of rice genes was performed using the Fisher's exact test implemented in a Perl script against the Kyoto Encyclopedia of Genes and Genomes (KEGG) database (Kanehisa et al., 2017).

### Network Analysis of Blast Resistance Genes

The main data on blast disease traits were collected from the China Rice Data Center (http://www.ricedata.cn/gene/gene_pi.htm). The blast resistance genes that were identified in the rice reference genome were used in the network analysis.

### Transcriptional Analysis of Rice Genes in Response to Blast Fungus

The RNA-seq datasets (accession: SRP079683) derived from rice with *M. oryzae* infection were downloaded from the NCBI SRA database (Bidzinski et al., 2016). The sequencing reads were processed by trimmomatic to remove the adapter sequences and low-quality reads (Bolger et al., 2014). The cleaned reads were mapped to the rice genome using hisat2 (Kim et al., 2019), and the aligned reads were counted with featureCounts (Liao et al., 2014). The differentially expressed genes were identified using DESeq2 with *p* < 0.01 and at least 2-fold changes (Love et al., 2014).

## Results

### Inference of Host–Pathogen PPIs From Structure Information

**Figure 1:**
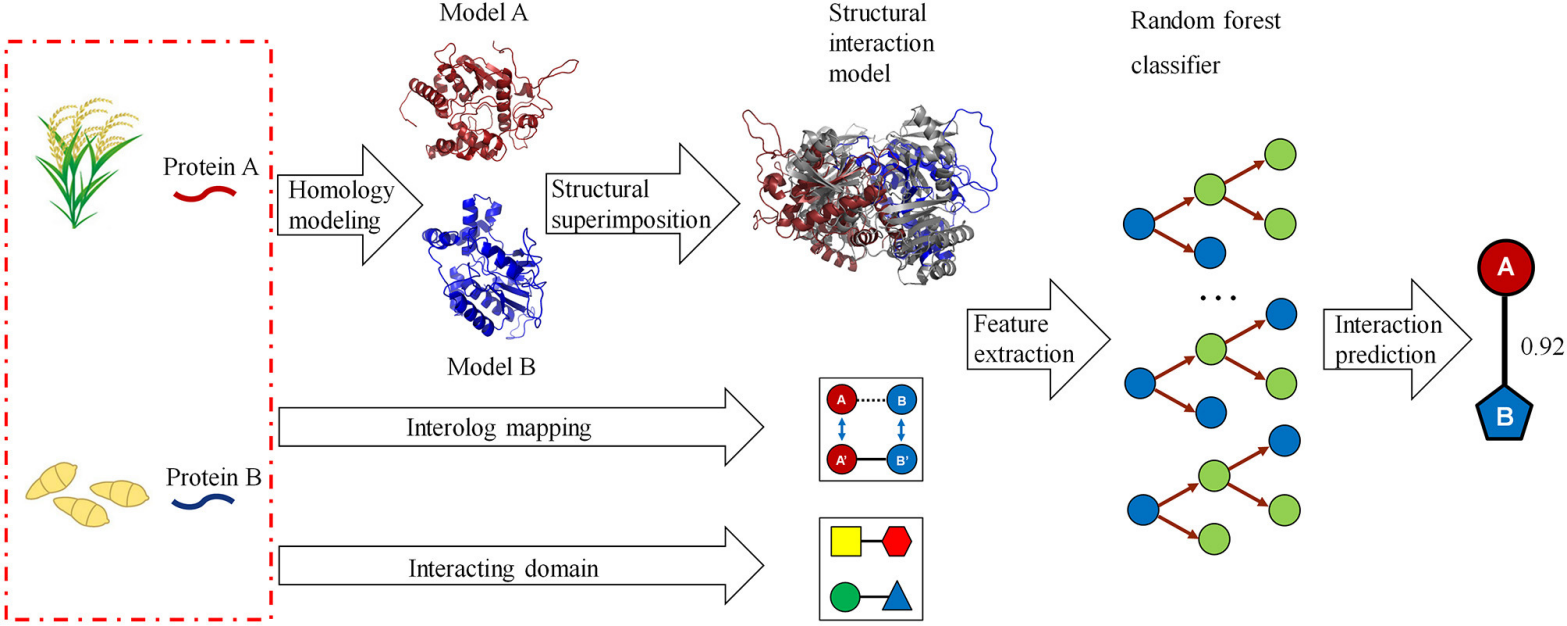
A computational framework for predicting protein–protein interactions (PPIs) between host and pathogen. Given a pair of potentially interacting proteins (A,B) from host and pathogen, three-dimensional structures for each protein pair were built by homology modeling and then searched for their closest PDB complex as a template by structural alignment. Four structure-based scores associated with the protein pair were calculated by superimposing the homology structures on their corresponding chains in the template complex. Finally, combining the structural evidences with the clues of homologous mapping and interacting domains to predict the interaction between proteins A and B using random forest-based classifiers.

We developed a computational method for predicting host–pathogen PPIs based on structural information. The framework is illustrated in Figure 1. In brief, given a pair of proteins from host and pathogen, we first predicted protein structures using homology modeling and then searched for their closest PDB complex as a structural template. The interaction model for each protein pair was created by superimposing the homology structures on their corresponding chains in the template complex. Structural features, including structural similarity, structural distance, as well as the number and fraction of the conserved interacting residue pairs, were calculated from the interaction model. Finally, we combined structural evidences and sequence information to predict host–pathogen PPIs using the random forest-based classifiers.

### Structure-Based Method Accurately Discovers Plant–Pathogen PPIs

**Figure 2:**
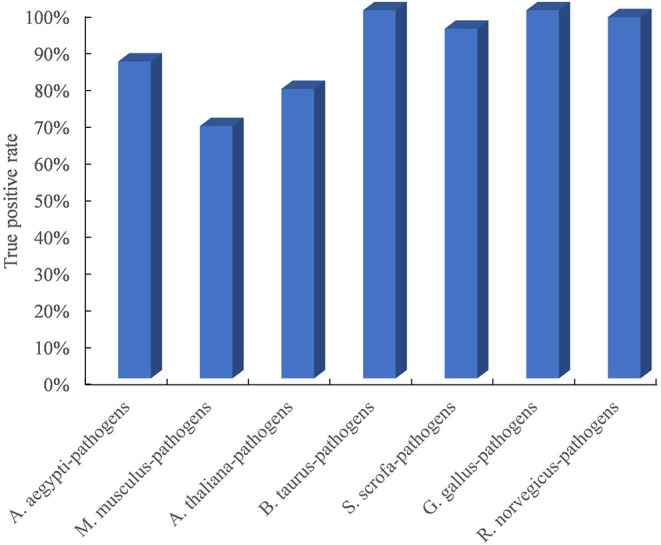
Performance evaluation of predicting PPIs across host–pathogen systems. The entire training dataset was partitioned into a test set and a training set. The test set contained the dataset from one selected host and all its pathogens, and the training set included all datasets related to the other remaining hosts and pathogens. For each test host–pathogen system, the process was performed 10 times with different negative training sets.

Due to the limited availability of known PPIs between plants and pathogens, cross-species performance is important for the model trained on the dataset in known host–pathogen systems to infer PPIs in a new host–pathogen system. We selected seven test host–pathogen systems to systematically evaluate model performance across host–pathogen systems. For each test, all datasets except from one selected host and all its pathogens were used to train the model, and the dataset from the selected host–pathogen system was used to evaluate the predictive model. As shown in Figure 2, the TPR of the method is higher than 68.6% for all test host–pathogen systems. The results indicated its robust cross-species prediction performance in discovering PPIs in new host–pathogen systems.

The interolog and domain-based methods are two widely used computational approaches for predicting host–pathogen PPIs. We thus evaluated the performance of our method with respect to these two methods on the test dataset of experimentally determined PPIs between *Arabidopsis* and all its pathogens. Among the 128 *Arabidopsis*–pathogen PPIs with homology-modeled structures, 101 (78.9%) protein interactions could be successfully predicted by the structure-based method. However, only 23 and 20 *Arabidopsis*–pathogen PPIs were inferred by the homologous mapping and interacting domain pairs, respectively (Table 1). For the interaction between *Arabidopsis* and *Ralstonia solanacearum*, 20 of PPIs were detected by the structural similarity, while only 4 of them were supported with the interacting domains (Supplementary Table 2). These results indicated that the structure-based method outperformed the interolog and domain-based methods for identifying plant–pathogen PPIs.

**Table 1:** The performance of different approaches in detecting protein–protein interactions (PPIs) between *Arabidopsis* and pathogen.

**Method**	**Accuracy (ACC)**	**True positive rate (TPR)**	**F1-score**
Structure-based method	224/256 = 87.5%	101/128 = 78.9%	0.86
domain-based method	148/256 = 57.8%	20/128 = 15.6%	0.27
interolog-based method	151/256 = 59.0%	23/128 = 18.0%	0.30

*The test dataset including 128 experimentally determined PPIs and 128 randomly shuffled protein pairs between Arabidopsis and pathogens*.

### Proteome-Wide Prediction of PPIs Between Rice and Blast Fungus

We first trained the prediction model on a dataset consisting of positive and negative examples of equal size. Although the model worked relatively well with an accuracy of 94.13%, the FPR of 5.37% would result in a large number of false positive interactions in the proteome-wide prediction of PPIs between rice and blast fungus. Thus, we reduced the FPR by expanding the size of the negative examples in the training dataset. When the ratio of positive to negative samples was adjusted to 1:70 in the training dataset, the FPR was decreased to the expected level of 0.10%, while the TPR remained at the relatively high level of 20.77% (Table 2).

**Table 2:** Performance comparison of models trained with different ratios of positive and negative samples.

**Positive:negative ratio**	**Accuracy (ACC) (%)**	**True positive rate (TPR) (%)**	**False positive rate (FPR) (%)**
1:1 (10148: 10148)	94.13	93.63	5.37
1:5 (10148: 50740)	93.09	84.14	5.12
1:10 (10148: 101480)	94.17	66.30	3.04
1:20 (10148: 202960)	96.16	42.79	1.17
1:30 (10148: 304440)	97.30	32.60	0.54
1:50 (10148: 507400)	98.32	23.84	0.19
1:60 (10148: 608880)	98.58	21.75	0.14
**1:70** (10148: **710360**)	**98.78**	**20.77**	**0.10**
1:80 (10148: 811840)	98.93	19.66	0.08
1:90 (10148: 913320)	99.04	18.90	0.07
1:100 (10148: 1014800)	99.13	18.30	0.06

*The bold values mean the optimized positive to negative ratio of training set with a relatively high TPR and a low level of FPR*.

To fill the gap between the number of protein sequences and 3D structures, protein structures for rice and blast fungus proteomes were predicted by using homology modeling. These predicted structures contained 32,170 and 2,910 models, covering 82.8 and 83.3% of rice and blast fungus secreted/transmembrane proteomes, respectively. Interaction models for 21,021,571 rice–blast fungus protein pairs were then created by superimposing the homology structures on their corresponding PDB templates.

**Figure 3:**
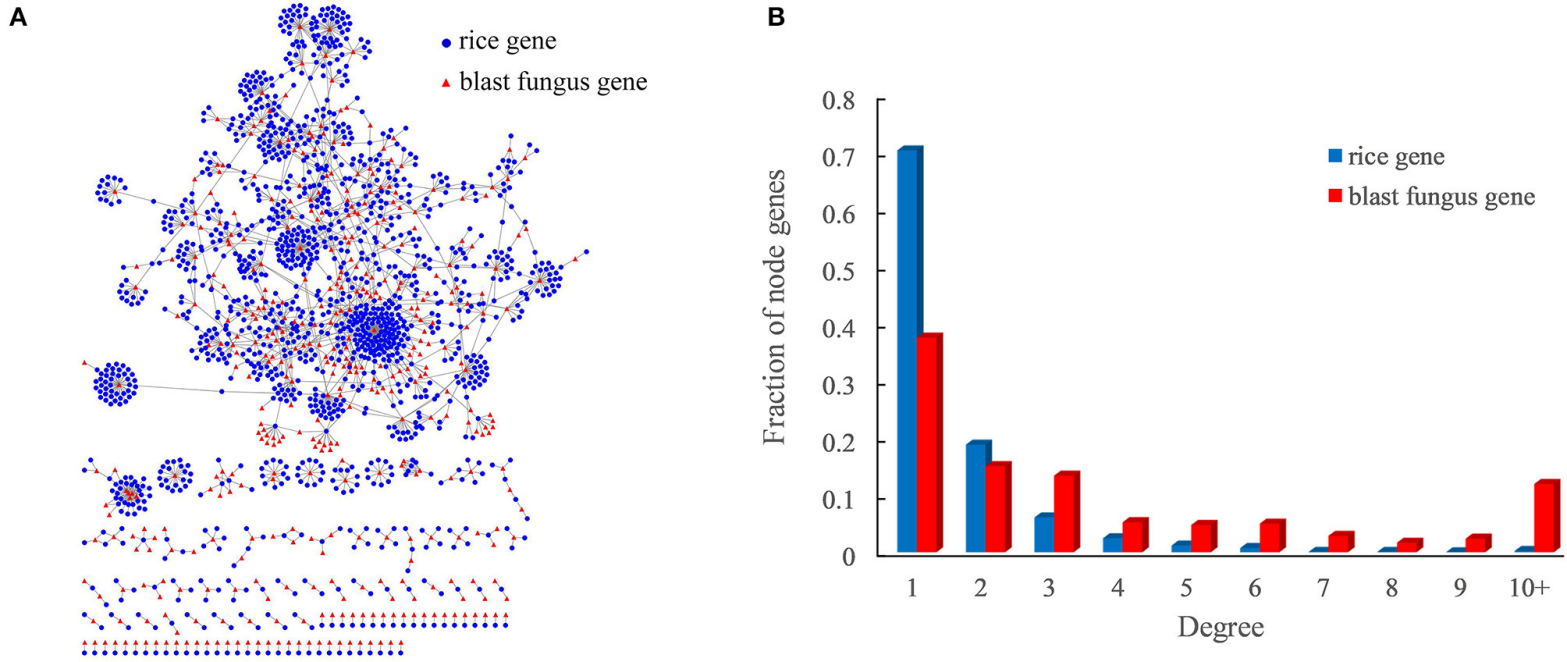
Rice–blast fungus PPI network. **(A)** Global view of the rice–blast fungus PPI network. Proteins from rice and blast fungus are represented by blue circles and red triangles, respectively. **(B)** Comparison between the degree distribution of rice and blast fungus proteins in the network. The blue bar represents the degree distribution of rice proteins interacting with blast fungus proteins, and the red bar represents the degree distribution of blast fungus proteins interacting with rice proteins.

The optimized host–pathogen prediction model was used to scan all rice–blast fungus protein pairs with the interaction models, resulting in a total of 2,018 PPIs between 1,344 rice proteins and 418 blast fungus proteins (Figure 3A, Supplementary Table 3). Of the predicted PPIs, only 29.9% (604) of interactions were supported with the evidences of sequence similarity and interacting domain pairs. Moreover, we found that 17 predicted PPIs could be identified by the previous study with the sequence-based method (Ma et al., 2019). These results indicated that the structure-based method could efficiently discover new rice–blast fungus PPIs beyond those interactions inferred from sequence similarity.

As expected, the rice–blast fungus PPI network exhibited scale-free properties similar to those of other biological networks (Supplementary Figure 1). It was interesting that the blast fungus proteins had more connections than rice proteins in the PPI network. One blast fungus protein averagely had five interacting partners from rice, while one rice protein interacted with around two blast fungus proteins (Figure 3B). Approximately, 12% (50) of blast fungus proteins had at least 10 rice interactors, and the pathogen protein with the highest degree was predicted to interact with 143 rice partners in the network. The result meant that the potential pathogen-associated proteins had a higher degree than the resistance-associated proteins in the rice–blast fungus PPI network.

### Network Captures Key Components in Rice–Blast Fungus Pathosystem

The major components involved in rice–blast fungus interactions include resistance genes from rice and avirulence effectors from *M. oryzae*. Thus, we examined whether the PPI network could predict these key components in rice–blast fungus pathosystem. Currently, about two dozens of blast resistance genes have been cloned and characterized in rice. Among the cloned resistance genes, 12 genes were distributed in the rice reference genome of *O. sativa* spp. *japonica* cv. Nipponbare (Supplementary Table 4). Two resistance genes, namely, pi-d2 (*LOC_Os06g29810*) and pi-ta (*LOC_Os12g18360*), were successfully predicted in the PPI network, ~7-fold enrichment in comparison with that of the whole genome. In addition, a total of 13 avirulence effector genes have been cloned from *M. oryzae*, six of which have the corresponding matches in the reference genome sequence (Supplementary Table 5). One avirulence effector, AVR-Pik (MGG_15972), was detected in the PPI network. The AVR-Pik effector was predicted to interact with four resistance-associated proteins (i.e., *LOC_Os02g37290, LOC_Os02g37300, LOC_Os02g37320*, and *LOC_Os04g39380*) containing a heavy metal-associated domain, which have been validated by experimental measurements (De la Concepcion et al., 2018, 2019). These results indicated the powerful performance of the PPI network in capturing major components in rice–blast fungus interactions.

### Functional Analysis of Rice Protein Involved in the Interaction

**Figure 4:**
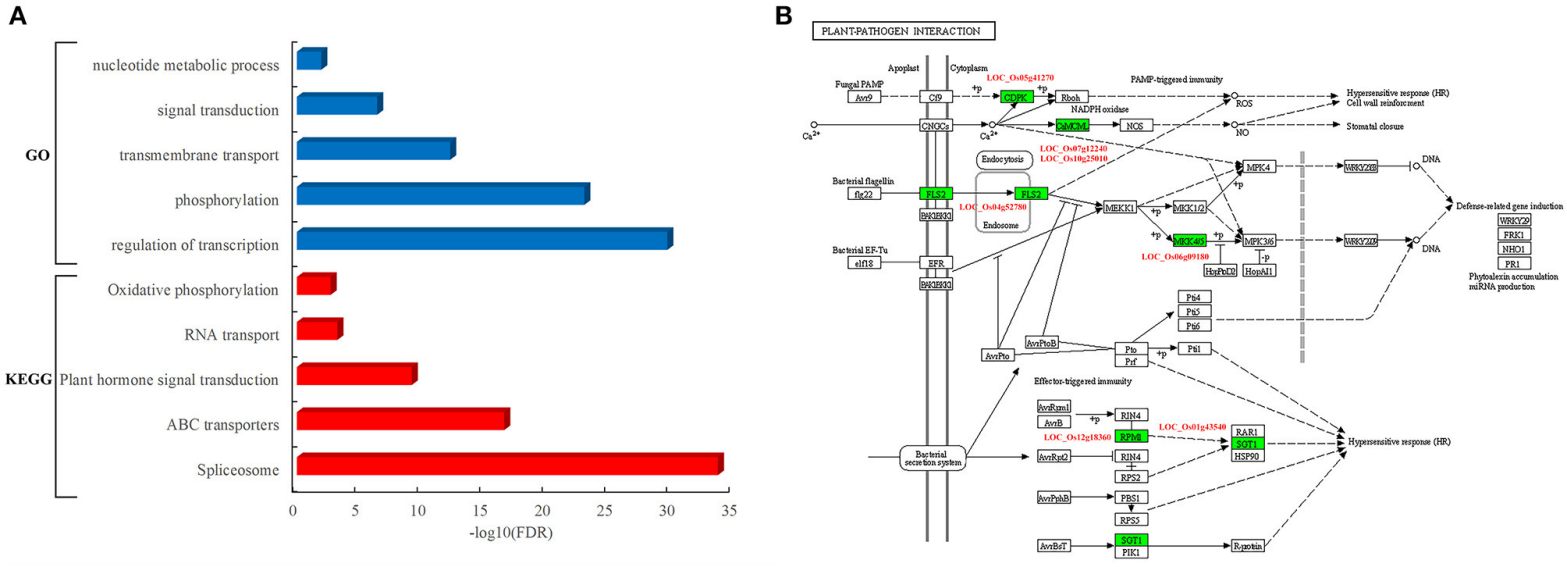
Functional and pathway analysis of rice genes in the PPI network. **(A)** Enriched function terms and pathways of rice genes. *Y*-axis represents the GO terms and KEGG pathways, and *X*-axis represents the negative log_10_(FDR) of enriched terms and pathways. The enriched GO terms and KEGG pathways are represented by blue and red bars, respectively. **(B)** Rice genes in the KEGG pathway of plant–pathogen interaction. The rectangle represents a gene product and the rectangle marked with green color indicates the corresponding rice gene as identified in the rice–blast fungus network.

To determine the function of rice proteins interacted with pathogen proteins, the GO analysis of these proteins in the network was carried out. These resistance-associated proteins were preferentially involved in specific biological processes such as transcriptional regulation, phosphorylation, transmembrane transport, signal transduction, and nucleotide metabolic process (Figure 4A). Furthermore, the pathway analysis showed that these rice proteins are significantly enriched in the pathways of spliceosome, ATP-binding cassette (ABC) transporters, plant hormone signal transduction, RNA transport, and oxidative phosphorylation (Figure 4A). It was worth to notice that seven rice proteins acted as core components in the pathway of plant–pathogen interactions, including *LOC_Os04g52780*, known as FLS2, an immune receptor, which could activate plant immune response by recognizing flagellin proteins of bacterial pathogens (Figure 4B).

### Network-Based Prediction of Blast Resistance Gene

**Figure 5:**
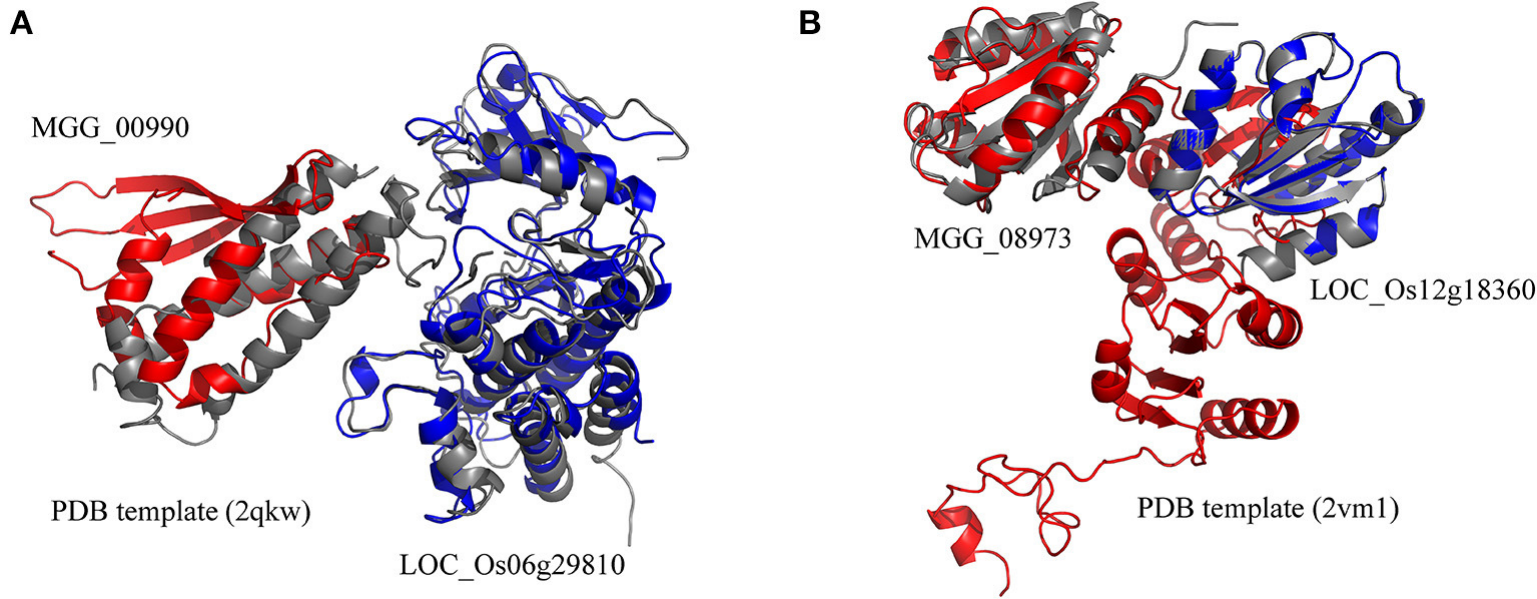
Structural model of protein interaction. **(A)** The structural interaction model for *LOC_Os06g29810* and *MGG_00990* created by superimposing the homology structures on the chains B and A in the template (PDB ID 2qkw). **(B)** The structural interaction model for *LOC_Os12g18360* and *MGG_08973* created by superimposing the homology structures on the chains A and C in the template (PDB ID 2vm1). The homology models of rice and blast fungus proteins are shown in blue and red, respectively. The PDB template complexes are shown in gray.

The rice blast resistance gene *LOC_Os06g29810* was predicted to interact with pathogen protein *MGG_00990* based on the structural interaction model created by superimposing the homology structures on the template of the AvrPto–Pto complex (Figure 5A). The homology model of *MGG_00990* was structurally similar to the pathogen effector AvrPto in the template complex, while the homology model of *LOC_Os06g29810* was structurally close to the plant resistance protein Pto (Xing et al., 2007). The another rice blast resistance gene *LOC_Os12g18360* could interact with the pathogen gene *MGG_08973*, which was inferred from the structural similarity of the homology models to the chains in the structural template of thioredoxin in barley (Figure 5B) (Maeda et al., 2008).

**Figure 6:**
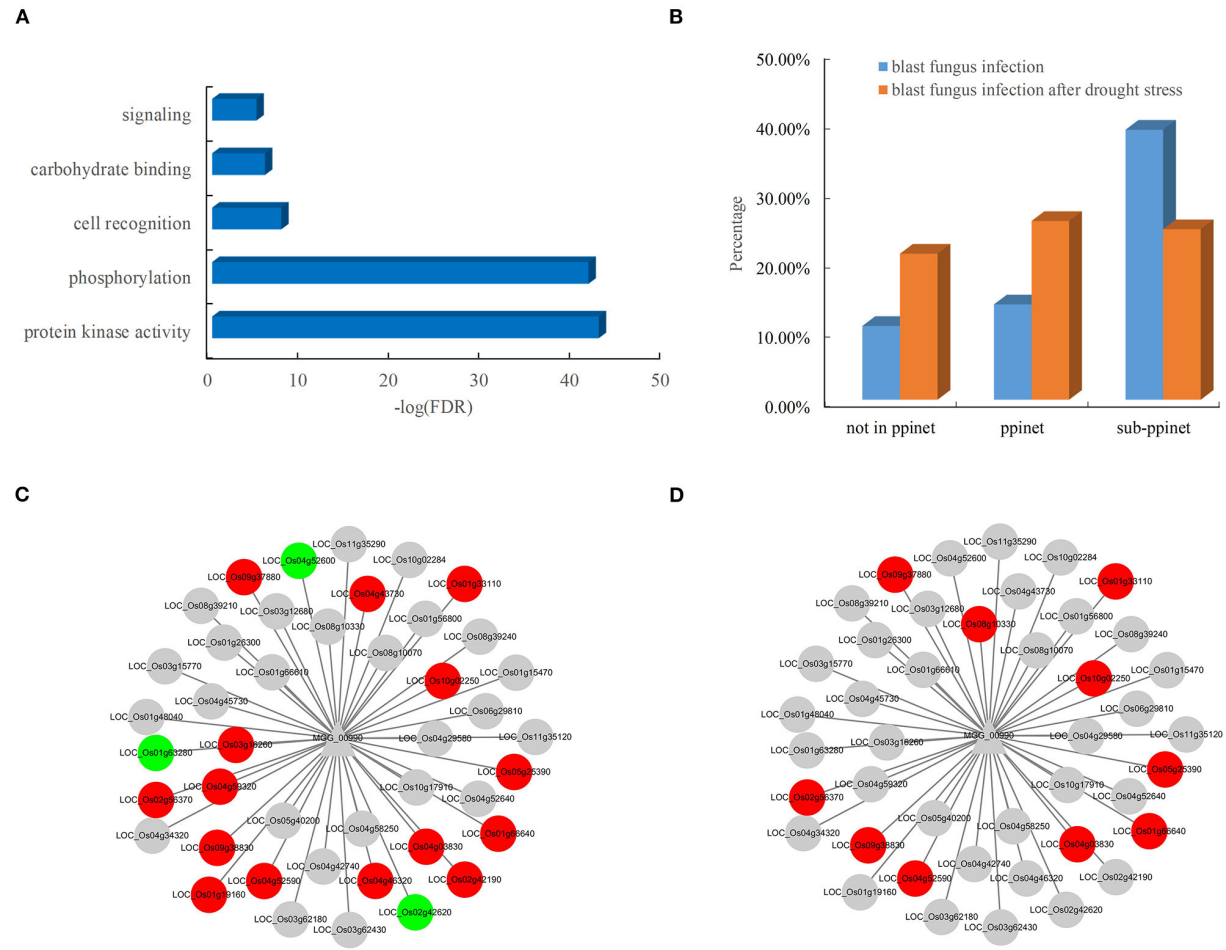
Functional significance and expression of rice genes in the subnet. **(A)** Enriched GO terms of rice genes in the blast resistance subnet. **(B)** Comparison of differentially expressed genes in rice after blast fungus infection. Rice genes in response to infection by **(C)** blast fungus and **(D)** blast fungus after drought stress in the subnet. The red nodes represent upregulated genes, and the green nodes represent downregulated genes.

The availability of the PPI network allowed the systematic discovery of novel blast resistance genes using the guilt-by-association method. In addition to the two blast resistance genes, 47 rice genes were found to interact with the two pathogen-associated genes, *MGG_00990* and *MGG_08973*, in the PPI network. The functional analysis revealed that these blast resistance candidates mostly encoded receptor-like cytoplasmic kinases involved in the biological processes of phosphorylation and signaling in rice (Figure 6A). Furthermore, the analysis of gene expression showed that these candidates were preferentially responsive to the infection of blast fungus (Figure 6B). Among the interacting partners of *MGG_00990*, 18 resistance-associated genes were differentially expressed after blast fungus infection (Figure 6C), while 10 genes were significantly induced by the infection blast fungus after drought stress (Figure 6D). These results indicated the good performance of the PPI network in discovering rice genes associated with blast resistance.

## Discussion

Determining PPIs is an essential step toward understanding the underlying biological mechanism against pathogen infection in plants (Muthamilarasan and Prasad, 2013). In this study, we presented a computational method for predicting plant–pathogen PPIs based on structural information. Using seven host–pathogen systems, we demonstrated that the structure-based method was powerful in discovering protein interactions across different host–pathogen systems. This advantage was of vital importance for machine learning-based method for predicting PPIs in new plant–pathogen systems based on prior knowledge obtained from known interacting proteins in other host–pathogen systems.

Although many experimentally validated host–pathogen PPIs have been deposited in the database, most of these PPIs (42,972 out of 45,200) focus on the protein interactions between humans and pathogens (Ammari et al., 2016). Due to the limited availability of plant–pathogen PPIs, many plant-specific pathogen effector proteins usually failed to identify any homolog in the known inter-species interactions using homologous sequence mapping (Yang et al., 2019). In addition, the intra-species PPIs from model organisms have been usually used to infer plant–pathogen PPIs (Li et al., 2012; Sahu et al., 2014). However, evolutionary differences between inter-species and intra-species PPI interfaces would limit the performance of plant–pathogen PPIs by transferring interactions across species (Franzosa and Xia, 2011). These weaknesses could be partially overcome by utilizing structural information. The developed method uses the structural similarity between proteins as a bridge to identify new interactions across plant–pathogen systems. Compared with the sequence-based methods, this structure-based approach enabled us to discover new interactions between plant and pathogen proteins that lacked significant sequence similarity with a known interaction template.

*Magnaporthe oryzae* is a notorious plant pathogen that causes the most destructive diseases of rice in the world. The prediction and analysis of PPIs are valuable in deciphering the molecular mechanisms of rice–blast fungus interactions. In this study, we generated a global rice–blast fungus PPI network that consisted of 2,018 interacting protein pairs involving 1,344 rice proteins and 418 blast fungus proteins. Over 70% of PPIs between rice and blast fungus were inferred from structural similarity, which greatly expanded the landscape of the rice–blast fungus PPI network. Compared with the previous PPI network, 17 of PPIs were identified by the structure-based method (Ma et al., 2019). Although the number of common PPIs was relatively small, the results were significantly overlapped between the two independent studies (i.e., Fisher's exact test *p* < 5.6e-39). Moreover, we noted that blast fungus proteins had more interacting partners than rice proteins in the network. Our findings were coherent with the sequence-based studies in which a few pathogen-associated proteins were involved in the plant–pathogen interactions (Li et al., 2012; Sahu et al., 2014; Ma et al., 2019). This is likely to be the result of the coevolutionary arms races, in which pathogens mutate genes extensively to infect their hosts, while plants defend against pathogen attacks by expanding gene families (Stahl and Bishop, 2000; Dangl and McDowell, 2006).

Despite the advances made in molecular mechanisms of rice resistance to blast fungus, many aspects of the rice immunity system remain obscure (Li et al., 2019). The rice–blast fungus PPI network showed that the AVR-Pik effector was successfully predicted to interact with four rice proteins, which have been validated by experimental approaches (De la Concepcion et al., 2018, 2019). In addition to the avirulence effector, two rice blast resistance genes were also identified in the network. Using the guilt-by-association method, we identified 47 candidate blast resistance genes in the PPI network. The majority of these genes that encoded receptor-like cytoplasmic kinases were involved in the response to the infection of blast fungus (Bidzinski et al., 2016). The PPI network provided a global map to help accelerate the identification of blast resistance genes and advance our understanding of the molecular mechanisms of plant–pathogen interactions.

## Data Availability Statement

The original contributions presented in the study are included in the article/Supplementary Material, further inquiries can be directed to the corresponding author/s.

## Author Contributions

CZ, LZhao, and LZhang designed the project and carried out the model training and computational validation. YL performed bioinformatic analyses. FS made substantial contributions to data collection. CZ, LZhao, and LZhang wrote the manuscript. All authors approved the manuscript.

## Conflict of Interest

The authors declare that the research was conducted in the absence of any commercial or financial relationships that could be construed as a potential conflict of interest.

## Publisher's Note

All claims expressed in this article are solely those of the authors and do not necessarily represent those of their affiliated organizations, or those of the publisher, the editors and the reviewers. Any product that may be evaluated in this article, or claim that may be made by its manufacturer, is not guaranteed or endorsed by the publisher.

## Supplementary Material

Supplementary Figure 1 Degree distribution of the node proteins.

Supplementary Table 1 Availability of protein–protein interactions (PPIs) in model organisms from various databases.

Supplementary Table 2 The experimentally determined PPIs between *Arabidopsis* and pathogens.

Supplementary Table 3 The predicted PPIs between rice and blast fungus.

Supplementary Table 4 The isolated dominant blast resistance genes in rice reference genome.

Supplementary Table 5 The isolated avirulence genes in *Magnaporthe oryzae* genome.
