# Supplementary figures and images for: Predicting Protein–Protein Interactions Between Rice and Blast Fungus Using Structure-Based Approaches
Source: Front Plant Sci. 2021 Jul 23;12:690124. doi: 10.3389/fpls.2021.690124 (PMC8343130; doi:10.3389/fpls.2021.690124)

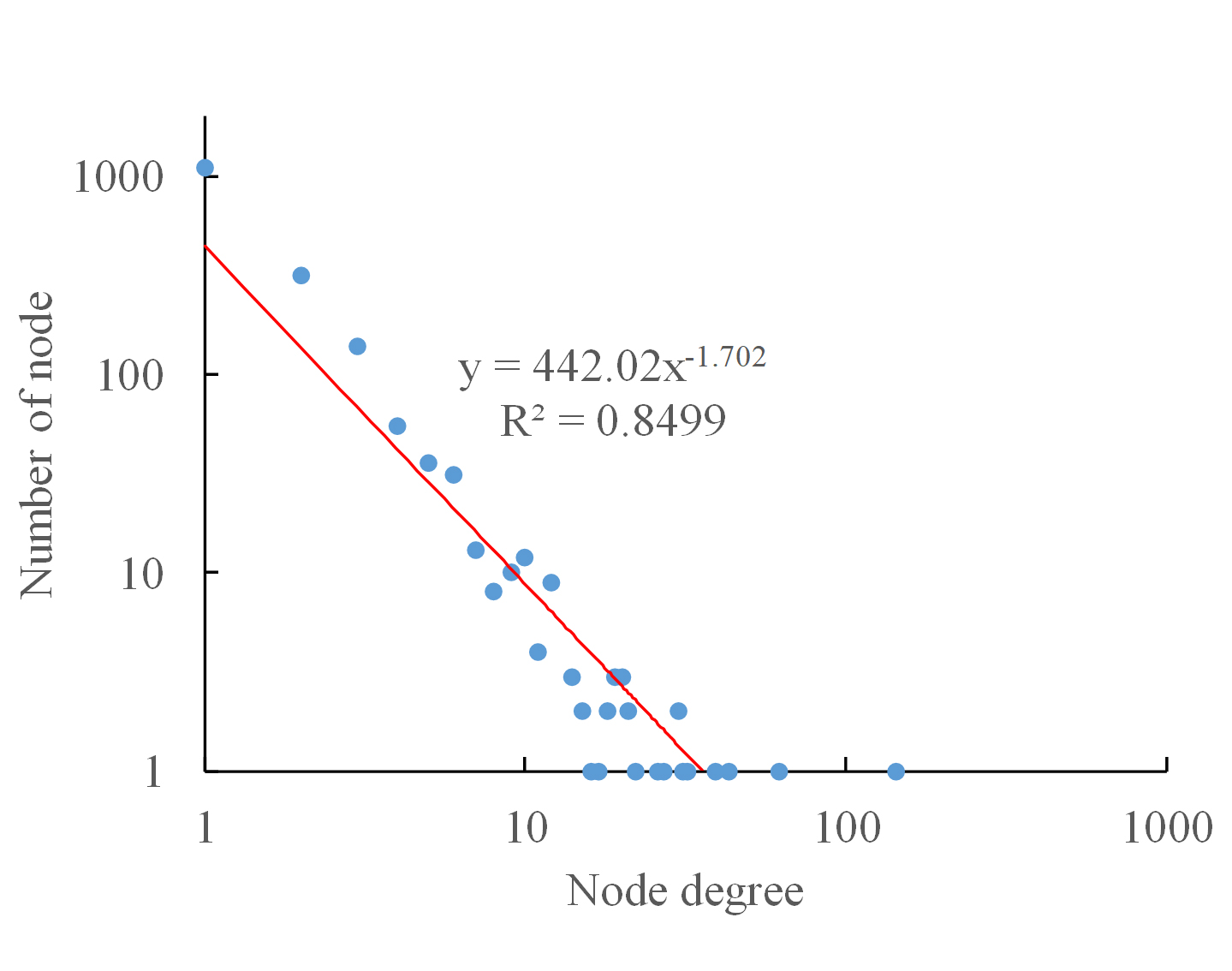

Supplement: Supplementary Figure 1 — Degree distribution of the node proteins. [file Image_1.JPEG]
