# Supplementary material for: Predicting Protein–Protein Interactions Between Rice and Blast Fungus Using Structure-Based Approaches
Source: Front Plant Sci. 2021 Jul 23;12:690124. doi: 10.3389/fpls.2021.690124 (PMC8343130; doi:10.3389/fpls.2021.690124)
Supplement: Supplementary Table 1 — Availability of protein–protein interactions (PPIs) in model organisms from various databases. [file Table_1.DOCX]

**Supplementary Table 1. Availability of PPIs in model organisms from various databases**

| Organism/Database | BioGRID | IntAct | DIP | BIND | MINT | Unique PPIs in total |
| --- | --- | --- | --- | --- | --- | --- |
| *H. sapiens* | 356934 | 256598 | 5168 | 19036 | 23369 | 480374 |
| ***M. musculus*** | 20945 | 22137 | 1344 | 2738 | 8443 | 41042 |
| *D. melanogaster* | 10957 | 30876 | 16000 | 15157 | 478 | 44446 |
| *C. elegans* | 18983 | 14266 | 2255 | 3601 | 422 | 31177 |
| *S. cerevisiae* | 110431 | 79199 | 0 | 22564 | 18627 | 170622 |
| *A. thaliana* | 48276 | 48276 | 466 | 977 | 404 | 52488 |
| *E. coli* | 0 | 17748 | 7311 | 74 | 203 | 20648 |
| All | 566526 | 469100 | 32544 | 64147 | 51946 | 840797 |
