# Supplementary material for: Predicting Protein–Protein Interactions Between Rice and Blast Fungus Using Structure-Based Approaches
Source: Front Plant Sci. 2021 Jul 23;12:690124. doi: 10.3389/fpls.2021.690124 (PMC8343130; doi:10.3389/fpls.2021.690124)
Supplement: Supplementary Table 4 — The isolated dominant blast resistance genes in rice reference genome. [file Table_4.DOCX]

**Supplementary Table 4. The isolated dominant blast-resistance genes in rice reference genome.**

| **Locus** | **Locus name** | **Gene** | **Prediction** |
| --- | --- | --- | --- |
| Pi-a | Magnaporthe oryzae resistance-a | LOC_Os11g11790 | No |
| Pi-b | Magnaporthe oryzae resistance-b | LOC_Os02g57310 | No |
| Pi5/Pi3/Pi-i | Magnaporthe oryzae resistance-5 | LOC_Os09g15840 | No |
| Pik-h/Pi-54 | Magnaporthe oryzae resistance-k | LOC_Os11g42010 | No |
| Pi-sh | Magnaporthe oryzae resistance-sh | LOC_Os01g57340 | No |
| Pi-t | Magnaporthe oryzae resistance-t | LOC_Os01g05620 | No |
| Pi-ta | Magnaporthe oryzae resistance-ta | LOC_Os12g18360 | Yes |
| Pi-9/Pi-2/Pi50 | Magnaporthe oryzae resistance-9 | LOC_Os06g17900 | No |
| Pi-d2 | Magnaporthe oryzae resistance-d2 | LOC_Os06g29810 | Yes |
| Pi-d3 | Magnaporthe oryzae resistance-d3 | LOC_Os06g22460 | No |
| pi-21 | Magnaporthe oryzae resistance-21 | LOC_Os04g32850 | No |
| Pi-56 | Magnaporthe oryzae resistance-56(t) | LOC_Os09g16000 | No |
