# Supplementary material for: Predicting Protein–Protein Interactions Between Rice and Blast Fungus Using Structure-Based Approaches
Source: Front Plant Sci. 2021 Jul 23;12:690124. doi: 10.3389/fpls.2021.690124 (PMC8343130; doi:10.3389/fpls.2021.690124)
Supplement: Supplementary Table 5 — The isolated avirulence genes in Magnaporthe oryzae genome. [file Table_5.DOCX]

**Supplementary Table 5. The isolated avirulence genes in *M. oryzae* genome.**

| **Avirulence gene** | **Genome locus** | **Description** | **Prediction** |
| --- | --- | --- | --- |
| Avr-Pita | MGG_15370 | Metalloproteinase | No |
| AVR-Pik | MGG_15972 | Uncharacterized protein | Yes |
| AvrPiz-t | MGG_18041 | Uncharacterized protein | No |
| Pwl2 | MGG_13863 | Uncharacterized protein | No |
| ace1 | MGG_12447 | Polyketide synthase-nonribosomal  peptide synthetase | No |
| AvrPi9 | MGG_12655 | Uncharacterized protein | No |
